# Benzalkonium Chloride Adaptation Increases Expression of the Agr System, Biofilm Formation, and Virulence in *Listeria monocytogenes*

**DOI:** 10.3389/fmicb.2022.856274

**Published:** 2022-02-23

**Authors:** Xiaobing Jiang, Congyi Jiang, Tao Yu, Xiaojie Jiang, Siyu Ren, Rui Kang, Shuxing Qiu

**Affiliations:** ^1^Henan Engineering Laboratory for Bioconversion Technology of Functional Microbes, College of Life Sciences, Henan Normal University, Xinxiang, China; ^2^School of Life Sciences & Basic Medicine, Xinxiang University, Xinxiang, China; ^3^Key Laboratory of Biomedicine and Health Risk Warning of Xinxiang City, Xinxiang, China

**Keywords:** *Listeria monocytogenes*, benzalkonium chloride, adaptation, the Agr system, biofilm formation

## Abstract

Benzalkonium chloride (BC) is widely used for disinfection in food industry. However, prolonged exposure to BC may lead to the emergence of BC adapted strains of *Listeria monocytogenes*, an important foodborne pathogen. Until now, two communication systems, the LuxS/AI-2 system and the Agr system, have been identified in *L. monocytogenes*. This study aimed to investigate the role of communication systems in BC adaptation and the effect of BC adaptation on two communication systems and the communication-controlled behaviors in *L. monocytogenes*. Results demonstrated that the Agr system rather than the LuxS system plays an important role in BC adaptation of *L. monocytogenes*. Neither *luxS* expression nor AI-2 production was affected by BC adaptation. On the other hand, the expression of the *agr* operon and the activity of the *agr* promoter were significantly increased after BC adaptation. BC adaptation enhanced biofilm formation of *L. monocytogenes*. However, swarming motility was reduced by BC adaptation. Data from qRT-PCR showed that flagella-mediated motility-related genes (*flaA*, *motA*, and *motB*) were downregulated in BC adapted strains. BC adaptation increased the ability of *L. monocytogenes* to adhere to and invade Caco-2 cells but did not affect the hemolytic activity. Compared with the wild-type strains, the expression levels of virulence genes *prfA*, *plcA*, *mpl*, *actA*, and *plcB* increased more than 2-fold in BC adapted strains; however, lower than 2-fold changes in the expression of hemolysis-associated gene *hly* were observed. Our study suggests that BC adaptation could increase the expression of the Agr system and enhance biofilm formation, invasion, and virulence of *L. monocytogenes*, which brings about threats to food safety and public health. Therefore, effective measures should be taken to avoid the emergence of BC adapted strains of *L. monocytogenes*.

## Introduction

Disinfection is an important operation in food industry, which can avoid the microbiological contamination of food products and reduce the risk of foodborne diseases (Pricope et al., 2013). Quaternary ammonium compounds (QACs) are widely used for disinfecting food processing environments. Benzalkonium chloride (BC) is one of the most studied quaternary ammonium disinfectants, and it exhibits good antibacterial activity against many important foodborne pathogens (Langsrud et al., 2003).

*Listeria monocytogenes*, the causative agent of listeriosis in humans and animals, represents a major foodborne pathogen. It can be found in a wide variety of raw and processed foods (Montero et al., 2015; Shamloo et al., 2019; Falardeau et al., 2021). Since *L. monocytogenes* has the ability to persist for long periods on equipments and within environments of food industry, the main contamination route for this pathogen is considered to be cross-contamination from food producing environments to food during processing (Mendonça et al., 2012; Jami et al., 2014; Smith et al., 2019). The recommended concentrations of BC for food industry are 200–1,000 μg/ml, which is high enough to inhibit the growth of *L. monocytogenes* completely (Møretrø et al., 2017). However, niches with sublethal concentrations of BC occur quite often due to improper use of BC, such as insufficient rinsing after disinfection and inadequate dosage (Møretrø et al., 2017). Previous studies have reported the emergence of adaptation to BC (it refers to “adaptive resistance to BC”) when *L. monocytogenes* are frequently exposed to sublethal concentrations of BC (Aase et al., 2000; Lundén et al., 2003; Romanova et al., 2006). Adaptation to BC can alter cell morphology, tolerance to environmental stresses, and sensitivity to antimicrobial agents in *L. monocytogenes* (To et al., 2002; Romanova et al., 2006; Bisbiroulas et al., 2011; Rakic-Martinez et al., 2011; Yu et al., 2018). To et al. (2002) have found that cells of BC adapted strains (it refers to “strains with adaptive resistance to BC”) were elongated and slimmer compared with the parent strains. In our previous study, we have reported decreased tolerance to acid, alkali, osmotic, ethanol, and oxidative stresses in BC adapted strains of *L. monocytogenes* (Yu et al., 2018). Besides, after BC adaptation, *L. monocytogenes* exhibits cross-adaptation not only to other disinfectants but also to antimicrobial agents, such as cephalosporin, ciprofloxacin, gentamicin, kanamycin, and ethidium bromide (Romanova et al., 2006; Rakic-Martinez et al., 2011; Yu et al., 2018).

Cell-to-cell communication is an important regulatory mechanism that allows bacteria to behave coordinately by producing and releasing chemical signal molecules. Two communication systems have been identified in *L. monocytogenes*, the autoinducer-2 (AI-2) LuxS and the autoinducing peptide (AIP) mediated Agr. The LuxS enzyme that involves biosynthesis of the signal molecule AI-2 is associated with the repression of biofilm formation of *L. monocytogenes* (Belval et al., 2006; Sela et al., 2006). The Agr system is composed of four genes (*agrB*, *agrD*, *agrC*, and *agrA*) organized as an operon, and it plays an important role in biofilm formation, virulence, and invasion in *L. monocytogenes* (Autret et al., 2003; Rieu et al., 2007; Riedel et al., 2009).

In the current study, we aimed to investigate (i) the role of two communication systems in BC adaptation, (ii) the effect of BC adaptation on expression of the communication systems, and (iii) the effect of BC adaptation on the communication-controlled behaviors, including biofilm formation and virulence in *L. monocytogenes*.

## Materials and Methods

### Bacterial Strains and Growth Conditions

Four previously characterized strains of *L. monocytogenes* (HL09, HL11, HL28, and HL50) were used in this study (Table 1). BC adapted strains of the four wild-type strains were also used in our study, and the sensitivity to BC of four adapted strains has been reported in our previous study (Yu et al., 2018). Each adapted strain carried the wild-type strain designation followed by BCA. Minimum inhibitory concentrations (MICs) of BC for all *L. monocytogenes* are presented in Table 1. BC adapted strains showed the similar growth curves as their parental strains when incubated in BHI broth at 37°C (data not shown). The increased resistance to BC of adapted strains remained stable for 1 week of daily subcultivation in BHI without BC (data not shown). *Listeria monocytogenes* strains were grown at 37°C on brain heart infusion (BHI; Oxoid Ltd., Basingstoke, Hampshire, England) agar or in BHI broth.

**Table 1:** *Listeria monocytogenes* strains used in this study.

Strain	Origin	Serotype	PFGE pattern	MIC of BC (μg/ml)
HL09	Cooked meat	4b	P13	6
HL09BCA				12
HL11	Cooked meat	1/2a	P3	6
HL11BCA				12
HL28	Chicken	1/2c	P4	4
HL28BCA				14
HL50	Cooked meat	1/2b	P12	4
HL50BCA				10

### Construction of Gene Deletion Mutant Strains

The gene deletion mutants of *luxS* and the entire *agr* operon derived from HL28 were generated by allelic replacement using a pMAD shuttle vector as described previously (Yu et al., 2018). Primers for construction of gene deletion mutants are listed in Table 2. In brief, an insert containing homologous arms up- and down-stream of the target gene was obtained by the splicing by overlap extension (SOE) PCR. The insert and pMAD were digested and ligated. The recombinant plasmid was electroporated into HL28 and transformants were seleced on BHI agar plates with erythromycin (5 μg/ml; Sigma-Aldrich, St. Louis, MO, United States).

**Table 2:** Primers used in this study.

Application	Gene	Primer name	Sequence (5’-3’)^a^
Mutant strain construction	*luxS*	lmo1288-1	*NNNNNN*ACGCGTTACAAACCACAACGCTACTCT (*Mlu*I)
		lmo1288-2	ACCTTCTAAACTATGGCTTGCTTTTTCTGCCATGCGTATC
		lmo1288-3	GATACGCATGGCAGAAAAAGCAAGCCATAGTTTAGAAGGT
		lmo1288-4	*NNNNNN*ACGCGTTTCCGCTTGATTCAGATACA (*Mlu*I)
	*agr*	lmo0048/0051-1	*NNNNNN*GGATCCAGAAGATGCAGGTGGAGTTG (*Bam*HI)
		lmo0048/0051-2	CTTTTGTCGTATCTAGCTCATG ATCATCTTTCCAGCGGTCT
		lmo0048/0051-3	AGACCGCTGGAAAGATGATCATGAGCTAGATACGACAAAAG
		lmo0048/0051-4	*NNNNNN*ACGCGT CGCTTCTTCTTCACTACGC (*Mlu*I)
RT-qPCR	16S rRNA	RT16S1	GGGAGGCAGCAGTAGGGA
		RT16S2	CCGTCAAGGGACAAGCAG
	*prfA*	RTlmo0200-1	AGAAACATCGGTTGGCTATT
		RTlmo0200-2	TTGACCGCAAATAGAGCC
	*plcA*	RTlmo0201-1	TACTCCCAGAACTGACACGA
		RTlmo0201-2	CTCGGACCATTGTAGTCATCT
	*hly*	RTlmo0202-1	TGACGAAATGGCTTACAGT
		RTlmo0202-2	TTTTCCCTTCACTGATTGC
	*mpl*	RTlmo0203-1	CGAATCGCTTCCACTCAC
		RTlmo0203-2	TTCGCATCGGTAAACTGG
	*actA*	RTlmo0204-1	CCTGTAAAGACCGCACCA
		RTlmo0204-2	GCTGATTCGCTTTCCTCTAC
	*plcB*	RTlmo0205-1	GACTGATTACCGAGAAGGG
		RTlmo0205-2	TGTCTTCCGTTGCTTGATA

a *Restriction sites are underlined. N, any of the bases*.

### Determination of MICs

The MICs of BC for *L. monocytogenes* were determined using the broth microdilution method as described previously (Romanova et al., 2006). Briefly, strains were tested in BHI broth using 96-well microtiter plates (Corning Inc., Kennebunk, ME, United States), with an inoculum of 10^4^–10^5^ CFU/ml. The plates were incubated at 37°C for 24 h. The lowest concentration of BC totally preventing growth was taken to be the MIC.

### Adaptation to BC

Adaptation to BC of *L. monocytogenes* was conducted as described previously (Aase et al., 2000). Briefly, strains were subcultivated in BHI containing BC at a concentration of 1/2MIC at 37°C from the start. Then subcultures were serially subcultivated in BHI with increasing BC concentration by steps of 0.5 μg/ml, until there was no growth within 7 days.

### Quantitative Real Time-PCR

In this study, the relative expression levels of *luxS*, *agrBDCA*, flagella gene (*flaA*), motility-related genes (*motA* and *motB*) and virulence-associated genes (*prfA*, *plcA*, *hly*, *mpl*, *actA* and *plcB*) were assessed by quantitative real time-PCR (qRT-PCR) as previously described (Jiang et al., 2021). qRT-PCR primers for six virulence genes are shown in Table 2, and the primers for *luxS*, *agr*, *flaA*, *motA* and *motB* have been reported in our previous study (Jiang et al., 2021).

### Detection of AI-2

A bioluminescence assay was used to detect AI-2 as described previously (Taga and Xavier, 2011). Briefly, overnight cultures of *L. monocytogenes* were centrifugated at 12,000 *g* for 10 min and the supernatants were filtered using a 0.22-μm-pore size filter (Millipore, Bedford, MA, United States). Then, the culture of *Vibrio harveyi* BB170 (an AI-2 reporter strain) and the filtered supernatants of *L. monocytogenes* were mixed at the ratio of 9:1 and incubated at 30°C. The fluorescence values of samples were measured by an EnSpire Multimode Plate Reader (PerkinElmer, Waltham, MA, United States). *Vibrio harveyi* BB170 and *Escherichia coli* DH5α were used as the positive control and the negative control, respectively. The relative activity of AI-2 was presented as a percentage of the positive control.

### Analysis of the *agr* Promoter (P_2_) Activity by β-Galactosidase Assays

The *agr* promoter (P_2_)-*lacZ* fusion was constructed as described previously (O’Driscoll et al., 2004; Collins et al., 2012). Briefly, the DNA fragment containing P_2_ was cloned into pPTPL, a promoter probe vector and then the recombinant plasmid was electroporated into the wild-type strains and the BC adapted strains. Transformants were selected by BHI agar plates with tetracycline (Sigma-Aldrich). β-galactosidase activity assay was performed based on the method by Miller (Deng et al., 2013). The assays were performed in triplicate independently, and results were presented as the mean in Miller unit.

### Microtiter Plate Biofilm Formation Assay

Biofilms were assayed using the microplate method with crystal violet staining as described previously (Djordjevic et al., 2002). Briefly, 200 μl of diluted (1:100 in BHI broth) bacterial culture (bacterial concentration of approximately 10^4^ CFU/ml) were transferred into microtiter plate (Corning). The plates were statically incubated at 37°C for 24 h, 48 h and 72 h. To assess the number of planktonic cells, the cultures (100 μl) were centrifuged, and the pellets were resuspended in 1 ml of sterile saline. The bacterial cultures were then serially diluted and 100 μl volumes were taken for colony counting. To quantify the biofilm production, the medium was removed after incubation and then the wells were gently washed five times with sterile water. Biofilms were stained with 1% crystal violet for 45 min and washed with sterile water. Finally, biofilms were decolorized by 95% ethanol. The absorbance at *OD*_595 nm_ was measured to determine biofilm production. Finally, the biofilms were visualized under a DMi1 inverted microscope (Leica-Microsystems, Wetzlar, Germany).

### Confocal Laser Scanning Microscopy Biofilm Formation Assay

Biofilm formation assay by confocal laser scanning microscopy (CLSM) was performed as described previously (Jiang et al., 2021). Biofilms were prepared by immersing the cover glasses in the wells of 24-well polystyrene plates (Corning). After 48 h of incubation at 37°C, biofilms were washed with sterile water. The Live/Dead BacLight Bacterial viability kit (Molecular Probes, Eugene, OR, United States) was used to stain biofilms. A Leica TCS-SP8 Confocal Laser Scanning Microscope (Leica-Microsystems) was applied for image acquisition. Three-dimensional projections of the biofilms were constructed from the CLSM acquisitions using the IMARIS 7.1 software (Bitplane, Zürich, Switzerland). The COMSTAT software was applied for quantification of biofilm biomass and thickness (Heydorn et al., 2000).

### Motility Assay

The swarming motility of all strains was tested on soft tryptic soy broth (TSB; Huankai Ltd., Guangzhou, Guangdong, China) agar plate (0.3% w/v agar) at 25°C and 37°C (Jiang et al., 2021). Bacteria were inoculated onto the agar plate using sterile toothpicks and the diameter of the bacterial swarm was measured 48 h later.

### Hemolysis Assay

The hemolytic activity of *L. monocytogenes* was assayed as described previously (Liu et al., 2016). The bacterial cultures were centrifuged (5,500 × *g*, 4°C, 10 min), and 250 μl of supernatant were mixed with 900 μl of hemolysin buffer (Solarbio Science & Technology, Beijing, China) and 100 μl of sheep red blood cells (Solarbio). Sterile BHI broth and the sheep red blood cells treated with 1% Triton X-100 (Solarbio) served as the negative control and positive control, respectively. The absorbance of the samples at 543 nm was measured and the relative hemolysis was determined as the percentage of the absorbance presented by the positive control.

### Caco-2 Adhesion and Invasion Assays

The ability of wild type and BC adapted strains to adhere to and invade Caco-2 cells was evaluated as described previously (Riedel et al., 2009). Briefly, bacterial cultures of *L. monocytogenes* were diluted in DMEM (Solarbio) to 1 × 10^8^ CFU/ml and then added to Caco-2 cells at a multiplicity of infection (MOI) of 100. The mixed cells were incubated at 37°C + 5% CO_2_ for 1 h. For adhesion assay, cells were washed with pre-warmed phosphate buffered saline (PBS; Solarbio) and then lysed with ice-cold distilled water. For invasion assay, cells were incubated in DMEM with 10 μg/ml gentamicin after washing once with pre-warmed PBS. The cells were washed and lysed according to the steps described above. The lysed cells were plated on BHI agar and incubated at 37°C for 24 h.

### Statistical Analysis

All data comparisons were analyzed using the unpaired two-tailed Student *t* test (Microsoft Excel 2010). Differences with values of *p* lower than 0.05 were considered as statistically significant.

## Results

### The Agr System Plays an Important Role in BC Adaptation

To investigate the role of two communication systems in BC adaptation of *L. monocytogenes*, the gene deletion mutant strains of *luxS* and *agr* derived from HL28 were constructed in this study. Our results displayed that deletion of *luxS* or *agr* had no effect on BC MICs for HL28 (Table 3). After BC adaptation, HL28Δ*luxS*BCA showed the MIC of BC with 14 μg/ml, the same value as that of HL28BCA; however, the BC MIC of HL28Δ*agr*BCA was lower than that of HL28BCA (Table 3). These results indicate that the Agr system rather than the LuxS system plays an important role in BC adaptation of *L. monocytogenes*.

**Table 3:** MICs of BC for *Listeria monocytogenes* strains.

Strain	MIC of BC (μg/ml)
HL28	4
HL28BCA	14
HL28Δ*luxS*	4
HL28Δ*luxS*BCA	14
HL28Δ*agr*	4
HL28Δ*agr*BCA	8

### Neither *luxS* Expression Nor AI-2 Production Was Affected by BC Adaptation

**Figure 1:**
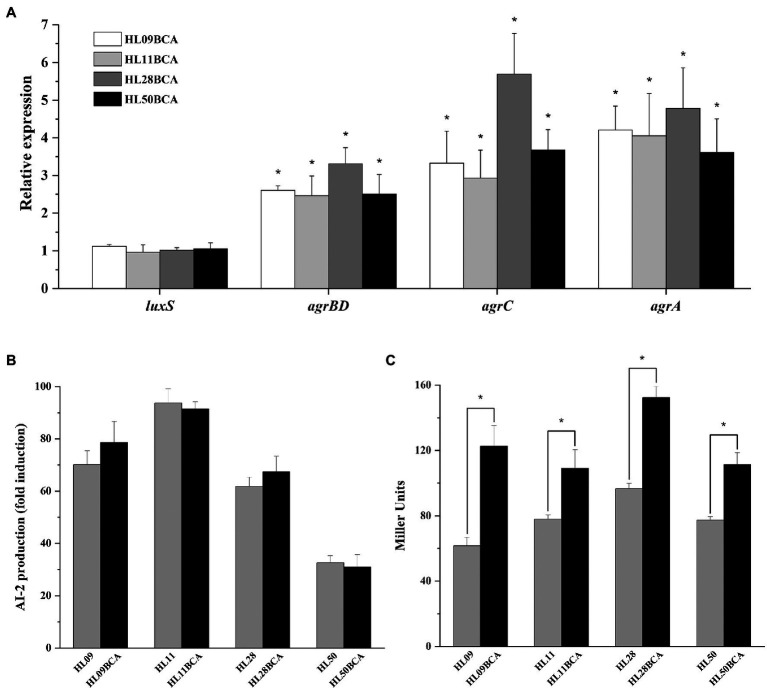
Effect of BC adaptation on **(A)** the expression levels of *luxS* and *agr*, **(B)** AI-2 production, and **(C)** the *agr* promoter (P_2_) activity. Relative expression levels of *luxS* and *agr* are presented as fold changes of the gene tested in BC adapted strain compared to that in its wild-type strain. Error bars represent the standard deviation of triplicate experiments (*n* = 3). The asterisk indicates a value statistically different from that of the wild-type strain, with *p* < 0.05.

Results from qRT-PCR showed that no obvious changes in the expression levels of *luxS* were observed in the adapted strains (Figure 1A). As presented in Figure 1B, four wild-type strains of *L. monocytogenes* had the ability to produce AI-2 and the highest level was observed in HL11. The amount of AI-2 of four BC adapted strains was similar to that of their corresponding wild-type strains.

### BC Adaptation Increases the Expression of the Agr System

The expression levels of *agr* genes in the BC adapted strains were significantly higher (*p* < 0.05) than those in their corresponding parental strains (Figure 1A). As shown in Figure 1C, the P_2_ activity of four BC adapted strains was increased significantly (*p* < 0.05) in relative to the corresponding wild-type strains. BC adaptation resulted in 1.99-, 1.40-, 1.58-, and 1.44-fold increase in the activity of the P_2_ promoter in HL09, HL11, HL28, and HL50, respectively.

### BC Adaptation Enhances Biofilm Formation of *Listeria monocytogenes*

**Figure 2:**
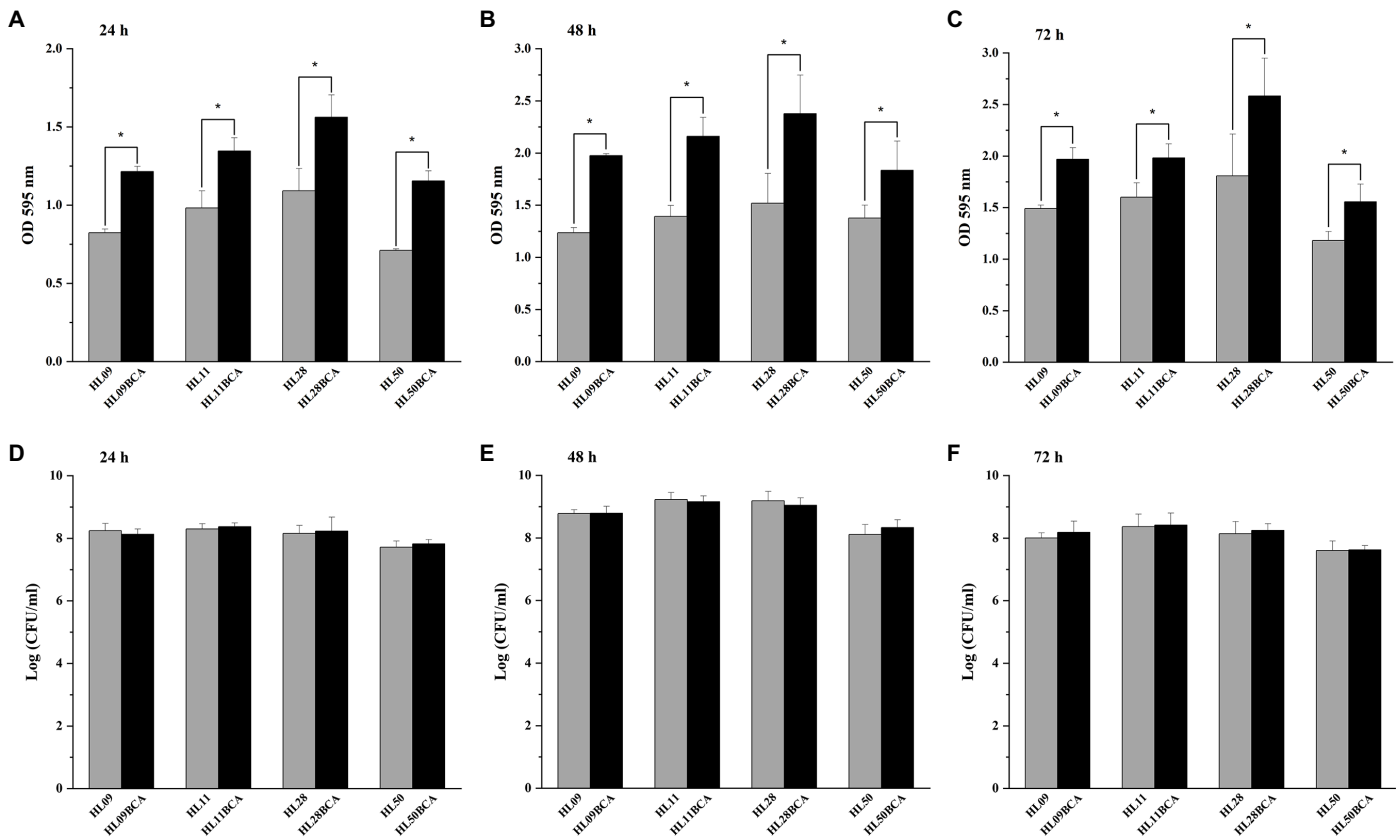
Biofilm formation assay by microtiter plate with crystal violet staining of the wild-type strains and the BC adapted strains of *Listeria monocytogenes* at 37°C for **(A)** 24 h, **(B)** 48 h, and **(C)** 72 h. Surviving planktonic cells in the bacterial culture incubated at 37°C for **(D)** 24 h, **(E)** 48 h, and **(F)** 72 h. Error bars represent the standard deviation of triplicate experiments (*n* = 3). The asterisk indicates a value statistically different from that of the wild-type strain, with *p* < 0.05.

The biofilm production of *L. monocytogenes* was quantified by the crystal violet staining method. As shown in Figures 2A–C, all the BC adapted strains demonstrated significantly increased biofilm biomass (*p* < 0.05) compared with their corresponding wild-type strains over each of 3 days at 37°C. For example, the biofilm biomass of HL09BCA was 47.5%, 60.0%, and 32.1% higher than that of HL09 at 24, 48, and 72 h, respectively. Each wild-type strain grew comparably with its BC adapted strain (Figures 2D–F). Therefore, the difference in biofilm formation is not due to a difference in growth.

**Figure 3:**
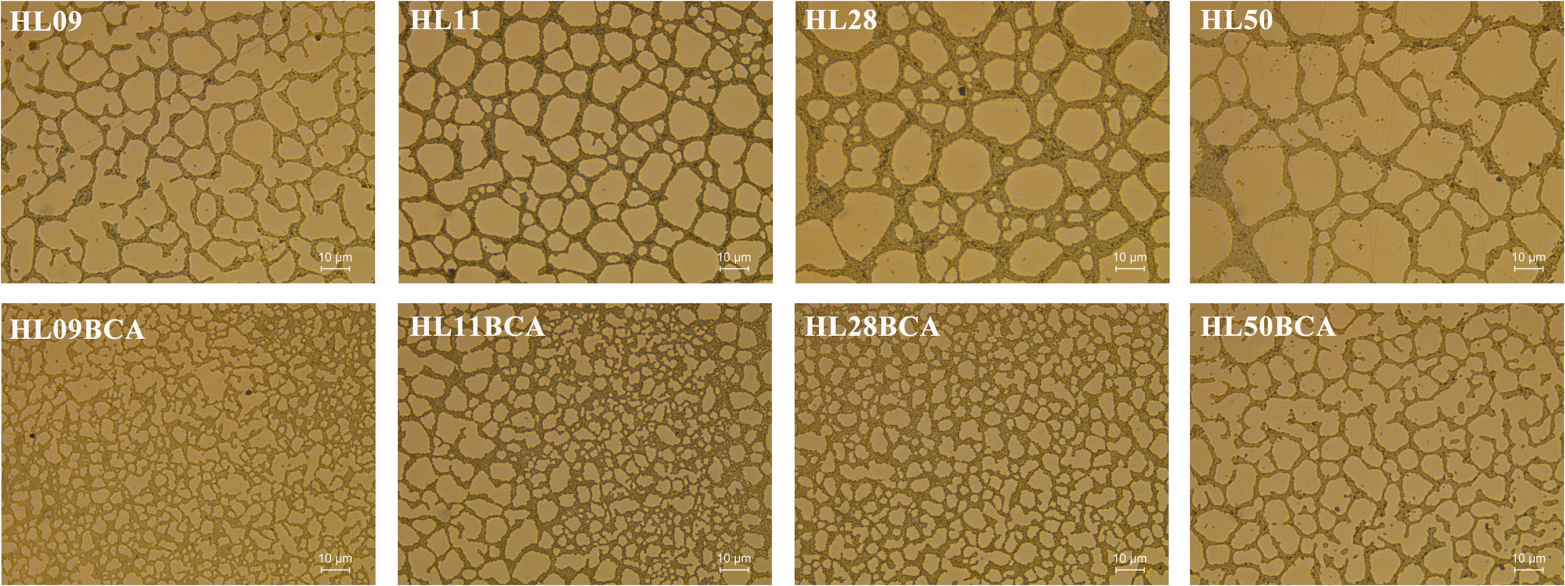
Inverted microscopic analysis of *Listeria monocytogenes* biofilm. All biofilms were grown at 37°C for 48 h.

**Figure 4:**
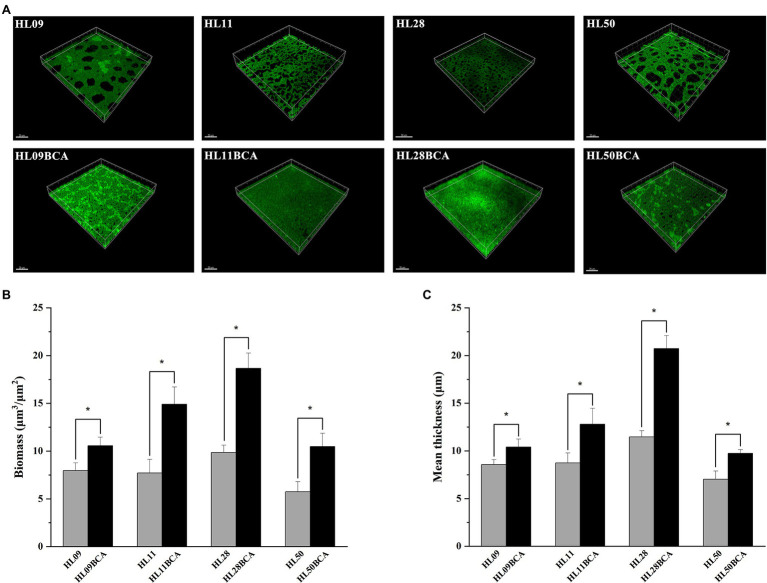
CLSM analysis of *Listeria monocytogenes* biofilm. **(A)** CLSM images of *Listeria monocytogenes* strains. **(B)** The biofilm biomass. **(C)** Mean thickness of biofilm. The asterisk indicates a value statistically different from that of the wild-type strain, with *p* < 0.05.

The morphology of *L. monocytogenes* biofilms was observed using inverted microscope and CLSM. Inverted microscopic images showed that compact biofilm structure with smaller pores was observed in the BC adapted strains, but loose biofilm structure with bigger pores was observed in their corresponding parental strains (Figure 3). CLSM showed that each BC adapted strain formed a denser and thicker biofilm than did its wild type (Figure 4). The results from microscopic analyses of biofilm formation were consistent with those from biofilm biomass determined by the microplate assay, indicating that biofilm formation of *L. monocytogenes* is enhanced after BC adaptation.

**Figure 5:**
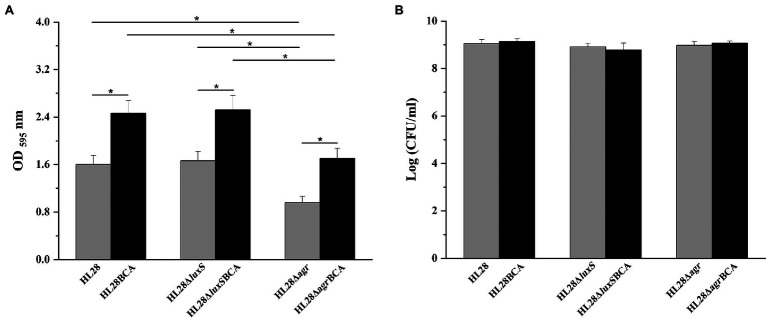
**(A)** Biofilm biomass of HL28, HL28BCA, HL28Δ*luxS*, HL28Δ*luxS*BCA, HL28Δ*agr* and HL28Δ*agr*BCA at 37°C for 48 h. **(B)** Surviving planktonic cells in the bacterial culture incubated at 37°C for 48 h. Error bars represent the standard deviation of triplicate experiments (*n* = 3). The asterisk indicates a value statistically different from that of the wild-type strain, with *p* < 0.05.

The biofilm-forming ability of four mutant strains HL28Δ*luxS*, HL28Δ*agr*, HL28Δ*luxS*BCA, and HL28Δ*agr*BCA was also investigated in our study. As shown in Figure 5, the absence of *luxS* had no influence on biofilm formation of HL28. HL28Δ*luxS*BCA exhibited a similar biofilm biomass as that of HL28BCA. Compared with the wild type strain HL28, the biofilm biomass was significantly reduced in the gene deletion mutant strain HL28Δ*agr* (*p* < 0.05). After BC adaptation, the biofilm biomass of HL28Δ*agr*BCA was higher than that of HL28Δ*agr* (*p* < 0.05) but still lower than that of HL28BCA (*p* < 0.05).

### BC Adaptation Reduces Swarming Motility

**Figure 6:**
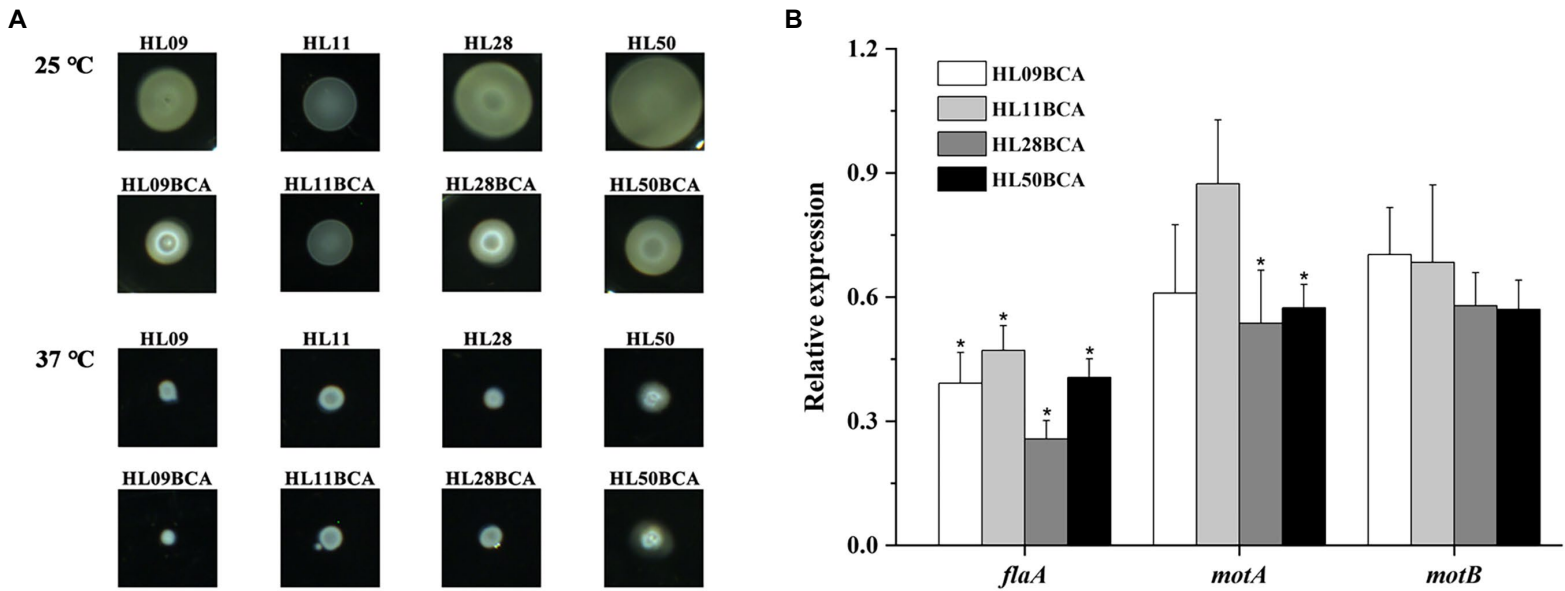
**(A)** Swarming motility of the wild-type strains and the BC adapted strains of *Listeria monocytogenes* at 25°C and 37°C. **(B)** Relative expression levels of flagella gene (*flaA*) and motility-related genes (*motA* and *motB*). The asterisk indicates a value statistically different from that of the wild-type strain, with *p* < 0.05.

In this study, we analyzed the motility of *L. monocytogenes* strains by semisolid swarm plate assays. After incubation at 25°C, all the strains were motile, and each BC adapted strain had a smaller swarm ring than that of its wild type (Figure 6A). At 37°C, none of the strains showed swarming (Figure 6A). Relative expression levels of flagella gene (*flaA*) and motility-related genes (*motA* and *motB*) were also measured by qRT-PCR. As shown in Figure 6B, the *flaA*, *motA*, and *motB* genes in the BC adapted strains were downregulated when compared to their corresponding wild-type strains, however, not all differences were significant. Expression levels of *flaA* in four BC adapted strains and *motA* in HL28BCA and HL50BCA were significantly decreased (*p* < 0.05). These data suggested that BC adaptation reduces the swarming motility of *L. monocytogenes*.

### BC Adaptation Increases Cell Adhesion and Invasion

**Figure 7:**
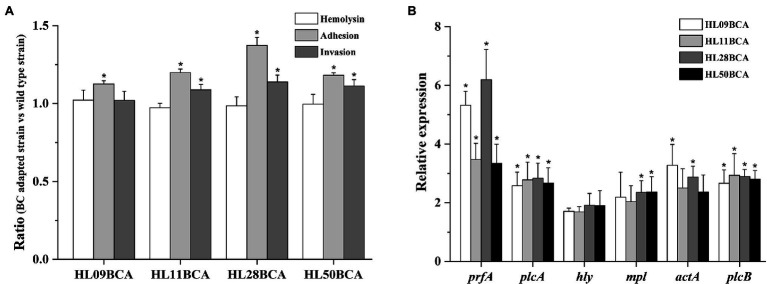
Effect of BC adaptation on **(A)** hemolysis, Caco-2 adhesion and invasion, and **(B)** expression of *prfA* virulence gene cluster. “Ratio” refers to the value of BC adapted strain relative to that of its wild type strain. Error bars indicate standard deviations. The asterisk indicates a value statistically different from that of the wild-type strain, with *p* < 0.05.

As presented in Figure 7A, there was no significant difference in the hemolytic activity between BC adapted strains and their corresponding wild type strains. The percentage of four BC adapted strains adhesion to Caco-2 cells was significantly higher (*p* < 0.05) than that of their corresponding wild-type strains. Specifically, BC adaptation increased the percent adherence by 12.6%, 19.9%, 37.3%, and 18.2% in HL09, HL11, HL28, and HL50, respectively. The percent invasion of HL11BCA, HL28BCA, and HL50BCA was increased by 8.79%, 13.9%, and 11.2%, respectively. No significant difference in the percent invasion was observed between HL09 and HL09BCA.

Expression levels of the *prfA* virulence gene cluster were investigated in this study (Figure 7B). Compared with the wild-type strains, the *prfA* gene was significantly upregulated (*p* < 0.05) in the BC adapted strains. The expression levels of *plcA*, *mpl*, *actA*, and *plcB* increased more than twofold; however, lower than twofold changes in expression of *hly* were observed in four adapted strains.

## Discussion

In the present study, the role of two communication systems in BC adaptation of *L. monocytogenes* was first investigated. The *luxS* gene and the *agr* operon were deleted from HL28, respectively. Since the mutants HL28Δ*luxS* and HL28Δ*agr* had the same BC MICs as that of HL28, these two systems may not be associated with resistance to BC. The same BC MICs were observed in HL28BCA and HL28Δ*luxS*BCA, indicating that BC adaptation was not affected by the absence of *luxS*. Compared with HL28BCA, HL28Δ*agr*BCA exhibited increased susceptibility to BC, suggesting the role of the Agr system in BC adaptation of *L. monocytogenes*. The Agr system is not the only mechanism of BC adaptation, because the BC MIC of HL28Δ*agr*BCA was twice that of HL28Δ*agr*.

To investigate the effect of BC adaptation on the communication systems, four strains of *L. monocytogenes* isolated from retail food with four different serotypes and their BC adapted strains were selected in our study. The LuxS/AI-2 system is ubiquitous in Gram-negative and Gram-positive bacteria and the production of AI-2 is dependent on LuxS, the key enzyme in the biosynthesis pathway of AI-2 (Sela et al., 2006). Our results suggested that BC adaptation affects neither *luxS* expression nor AI-2 production.

The *agr* system in *L. monocytogenes* consists of the four-gene operon *agrBDCA*. Of the four proteins encoded by the *agr* operon, a precursor peptide AgrD is processed into an active signaling molecule AIP by AgrB and then the AIP is released outside the cells (Autret et al., 2003; Garmyn et al., 2009). Upon accumulation in the extracellular space, the AIP activates the two-component system consisting of AgrC (receptor-histidine kinase) and AgrA (response regulator; Autret et al., 2003; Garmyn et al., 2009). Our results showed that BC adaptation increased the expression of the *agr* operon. The putative promoter of the *agr* operon, the P_2_ promoter, has been found upstream of *agrB* in *L. monocytogenes* (Autret et al., 2003). In our study, the effect of BC adaptation on the P_2_ promoter activity was also investigated. After BC adaptation, the activity of P_2_ was significantly increased in four tested strains, providing further evidence that BC adaptation could activate the transcription of the Agr system.

*Listeria monocytogenes* is capable of forming biofilm on various food processing surfaces. Biofilms by *L. monocytogenes* represent an important source of contamination of raw materials and processed products, which brings about a great potential threat to food safety. Given that the Agr system positively regulates biofilm formation of *L. monocytogenes* (Rieu et al., 2007; Riedel et al., 2009), a promotion of Agr may enhance the development of bacterial biofilms. BC adaptation has been confirmed to induce the Agr system in this study. Thus, we speculated that BC adaptation also affects biofilm formation of *L. monocytogenes*. Our results demonstrated that the biofilm-forming ability of BC adapted strains was much stronger than that of wild-type strains, suggesting that BC adaptation could enhance biofilm formation mediated by the Agr system in *L. monocytogenes*.

Romanova et al. (2007) assessed biofilm formation of three *L. monocytogenes* strains and their BC adapted strains by crystal violet staining, and they found that the adaptation to BC does not significantly affect biofilm-forming ability. Their findings were different from our results, which may be caused by different experimental protocols. In the previous study, biofilm was incubated at 30°C for 5 days and the medium was replaced with fresh broth every 24 h (Romanova et al., 2007). In our study, biofilm was incubated at 37°C for 24, 48, and 72 h, and the medium was not changed during the incubation period. Additionally, biofilm biomass was determined by measuring the absorbance at 530 nm and the observed *OD*_530 nm_ values were in the range from 0.03 to 0.17 in the study of Romanova et al. (2007), which were much lower than our *OD*_600 nm_ values.

Previous studies have reported that the *luxS* deletion mutant strain exhibited an enhanced ability to form biofilm compared with the wild type strain of *L. monocytogenes* EGD-e (Belval et al., 2006; Sela et al., 2006). However, the absence of *luxS* had no influence on biofilm formation of HL28 in our study. The different results may be due to the different strains used. Our results also showed the biofilm biomass of HL28Δ*agr* reduced when compared with that of HL28, providing further evidence for the positive regulation of Agr on biofilm formation of *L. monocytogenes*. The biofilm forming ability of HL28Δ*luxS*BCA and HL28Δ*agr*BCA was higher than that of HL28Δ*luxS* and HL28Δ*agr*, confirming that BC adaptation could enhance biofilm formation of *L. monocytogenes*.

Many studies have confirmed the importance of flagellum-mediated motility in the first stages of biofilm formation (Shrout et al., 2011; Guttenplan and Kearns, 2013). Swarming motility is a specialized form of movement and enables flagellated bacteria to coordinately move atop solid surfaces (Fraser and Hughes, 1999). Increased swarming motility may improve biofilm-forming ability. The flagellum of *L. monocytogenes* is composed of flagellin monomers encoded by the *flaA* gene. The *motA* and *motB* genes encode the flagellar motor protein MotA and the flagellar motor rotation MotB, respectively. Previous studies have reported that these flagellar motility genes (*flaA*, *motA*, and *motB*) are critical for *L. monocytogenes* biofilm formation (Lemon et al., 2007; Todhanakasem and Young, 2008). In this study, BC adaptation reduced not only swarming motility but also the expression levels of *flaA*, *motA* and *motB*. Our results suggested that BC adaptation had opposite effects on swarming motility and biofilm formation. Although swarming motility is considered as one of the factors affecting biofilm formation, there is no clear positive correlation between them (O’May et al., 2012; Henly et al., 2019). Thus, it was not surprising that BC adapted strains exhibited enhanced biofilm formation and decreased swarming motility.

*Listeria monocytogenes* is an intracellular pathogen that can cause severe invasive infections mainly in the newborn, the elderly, and the immunocompromised. Actually, the process of *L. monocytogenes* infecting host cells is under the modulation of virulence factors (Camejo et al., 2011). The virulence gene cluster composed of *prfA*, *plcA*, *hly*, *mpl*, *actA*, and *plcB* has been identified in *L. monocytogenes*, the products of which play a crucial role in pathogenesis (Sheehan et al., 1995). Among these genes, *hly* encodes listeriolysin O (LLO), which is a Listeria-specific hemolysin and major virulence factor required for escape of bacteria from the phagocytic compartment into the cytoplasm (Sheehan et al., 1995). All the genes in this virulence gene cluster are controlled by the transcriptional activator PrfA (Sheehan et al., 1995). Previous studies reported that the deletion of *agrD* resulted in reduced virulence and expression of *prfA*-dependent virulence gene cluster, indicating that Agr system is involved in virulence mediated by this gene cluster (Riedel et al., 2009). Our results found that BC adaptation increased the ability of *L. monocytogenes* to adhere to and invade Caco-2 cells. Indeed, *prfA* and several *prfA*-controlled virulence genes were upregulated in BC adapted strains. However, the hemolysis activity of *L. monocytogenes* was not affected by BC adaptation. The expression levels of *hly* were not significantly changed in BC adapted strains. There is the possibility that *hly* is controlled by other regulator(s).

Previous studies reported that the sublethal concentrations of BC inhibited biofilm formation and Caco-2 cell invasion of *L. monocytogenes* (Pricope et al., 2013; Ortiz et al., 2014). In the current study, results suggested that BC adaptation increased the ability to form biofilms and invade Caco-2 cells in *L. monocytogenes*. These findings are not contradictory because BC adaptation is different from BC exposure. Specifically, prolonged exposure to BC is required for *L. monocytogenes* strains to develop adaptive tolerance to this disinfectant. Compared with its wild-type strain, BC adapted strain could exhibit changes in cell morphology, environmental stress response, antimicrobial resistance, and so on. These phenotype changes may be due to mutations.

The Agr system plays an important role in BC adaptation of *L. monocytogenes*. BC adaptation promotes the Agr system of *L. monocytogenes* and consequently enhances bacterial behaviors controlled by this communication system. BC adapted strains of *L. monocytogenes* exhibited increased ability to form biofilm and adhere to and invade Caco-2 cells. Therefore, the presence of BC adapted strains in food processing environments may increase the risk of food contamination by *L. monocytogenes* and bring about threats to food safety and public health. Our study indicates the importance of proper use of disinfectants. The operators should use the disinfectant BC according to standardized protocols in food industry, which may reduce bacterial exposure to sublethal concentrations of BC and the emergence of BC adapted strains.

## Data Availability Statement

The raw data supporting the conclusions of this article will be made available by the authors, without undue reservation.

## Author Contributions

XjJ, CJ, SR, and RK performed the experiments. XbJ analyzed the data and drafted the manuscript. TY and SQ designed and supervised the study. All authors contributed to the article and approved the submitted version.

## Funding

This work was supported by the Cultivation Fund of the National Scientific Research Project of Henan Normal University (2020PL04), the Natural Science Foundation of Henan Province (202300410018), and the Key Project of Natural Science of the Education Department of Henan Province (22A180003).

## Conflict of Interest

The authors declare that the research was conducted in the absence of any commercial or financial relationships that could be construed as a potential conflict of interest.

## Publisher’s Note

All claims expressed in this article are solely those of the authors and do not necessarily represent those of their affiliated organizations, or those of the publisher, the editors and the reviewers. Any product that may be evaluated in this article, or claim that may be made by its manufacturer, is not guaranteed or endorsed by the publisher.
